# Numbers and Values of Farm Animals in the United States

**Published:** 1890-04

**Authors:** 


### NUMBERS AND VALUES OF FARM ANIMALS IN THE UNITED STATES.

THE report of the statistician of the Department of Agriculture, Report No. 70, Washington, January and February, 1890, furnishes us with valuable figures of interest, both to ourselves, as veterinarians, and to those to whom we are most intimately related, the stock breeder, the agriculturist and the consumers of horses and other live stock.

14,213,837 horses is an increase of 550,543 horses over last year, but the total value, $978,516,562, is a decrease of $3,678,265

since the same period. The current valuation is $68.84 per head, against $71.89 last year, $71.82 two years ago, and an average of $72.58 for the past six years. The report remarks: "This indicates a full supply, and mildly points to a possible tendency to over-production and lower prices, furnishing a point which horsemen will do well to study.

A gain of 73,453 mules brings the number to 2,331,027, valued at $182,394,099, an increase of $2,949,618.

15,952,883 milch cows is a gain of 654,258, and 36,849,024 oxen and other cattle a gain of 1,816,607 animals. But the valuation of $353,152,133 for milch cows, and $560,625,137 for oxen and other cattle, shows a respective decrease of $13,074,243 and $36,611,675. The decrease of valuation with increase of the numbers of cattle is due to a falling off in price of $1.80 per head for milch cows, now valued at $22.14, and $1.84 per head for oxen and other cattle, now valued at $15.21. Notwithstanding the growth in numbers there is an aggregate decrease in value of a little over 5 per cent. No other commentary is needed on the worst cattle year the country has ever known.

Sheep show an increase in numbers for the first time since 1884—1,736,993 of a gain—making the present total 44,336,072 head. This increase in number is accompanied, too, by an increase of over 6½ per cent. in per capita value, or a change from $2.13 to $2.27 per head. Sheep are higher now than in any year since 1884, the average value for the five intervening years being about $2.05 per head. Considering the adverse conditions affecting the wool market the sheep industry has done at least as well as could have been expected, and it shows at the present time an excellent tone.

The country has 1,301,188 more swine than a year ago, the present aggregate being 51,602,780 head, valued at $243,418,336, a decrease of $47,888,857, the current $4.72 per head being $1.07 lower than a year ago. Notwithstanding this decline there is nothing especially discouraging in the outlook for swine. The average value for ten years has been $5.22, and it has been an unusually good ten years in swine prices.

Taking the live stock of the country in the aggregate, 165,285,623 animals, we find nothing except mules and sheep which have increased in total value within the last year. All kinds of stock taken into the count, the valuation is placed at $2,418,766,-

028—a fall-off of 3½ per cent. ($88,284,030), from the valuation of January, 1889. Estimating the population of the United States at 65,000,000, and 44 per cent. of this as rural, there would be an average of about $8.45 worth of live stock for each individual living on a farm.

It is not often that the *per capita* distribution of live stock would show as low a figure, and we question whether it will again for a number of years, and seek a solution for the present apparent depression in prices. In the markets of the better agricultural regions and the large cities the answer will be readily found.

Well-bred roadsters and coach horses, and sound, good horses for light or heavy draft work, are bringing better prices than they ever did before, but they are difficult to find, while the large numbers of common animals brought to market will only find a sale at the lower figures, which our more expert buyers have learned are all they are worth.

The sales of Shorthorns, Herefords, Friesians and all the better class of pure-bred cattle show a steady increase in prices, while the decrease is found in the medium and inferior animals. When all breeders will learn that sound horses, well bred for a specific object, out of sound horses and mares, which fulfilled what was wanted, will always bring large prices, and that defective and wanting animals cost just as much to raise, and will always be bought with suspicion ; when the farmer will learn that well-bred, large cattle dress 5 to 10 per cent. more beef than common, small cattle, and always bring prices greater in proportion than the increase in their weight, and breed accordingly, then the value of our animal industry will make a great increase.

